# Treatment of Nasopharyngeal Carcinoma Cells with the Histone-Deacetylase Inhibitor Abexinostat: Cooperative Effects with Cis-platin and Radiotherapy on Patient-Derived Xenografts

**DOI:** 10.1371/journal.pone.0091325

**Published:** 2014-03-11

**Authors:** Mélanie Gressette, Benjamin Vérillaud, Anne-Sophie Jimenez-Pailhès, Hélène Lelièvre, Kwok-Wai Lo, François-Régis Ferrand, Charles-Henry Gattolliat, Anne Jacquet-Bescond, Laurence Kraus-Berthier, Stéphane Depil, Pierre Busson

**Affiliations:** 1 Université Paris-sud, CNRS-UMR 8126 and Gustave Roussy, Villejuif, France; 2 Institut de Recherches Internationales Servier, Suresnes, France; 3 The Chinese University of Hong-Kong, Hong Kong SAR, China; 4 Hôpital du Val de Grâce, Paris, France; Karolinska Institutet, Sweden

## Abstract

EBV-related nasopharyngeal carcinomas (NPCs) still raise serious therapeutic problems. The therapeutic potential of the histone-deacetylase (HDAC) inhibitor Abexinostat was investigated using 5 preclinical NPC models including 2 patient-derived xenografts (C15 and C17). The cytotoxicity of Abexinostat used either alone or in combination with cis-platin or irradiation was assessed *in vitro* by MTT and clonogenic assays using 2 EBV-negative (CNE1 and HONE1) and 3 EBV-positive NPC models (C15, C17 and C666-1). Subsequently, the 3 EBV-positive models were used under the form of xenografts to assess the impact of systemic treatments by Abexinostat or combinations of Abexinostat with cis-platin or irradiation. Several cell proteins known to be affected by HDAC inhibitors and the small viral non-coding RNA EBER1 were investigated in the treated tumors. Synergistic cytotoxic effects of Abexinostat combined with cis-platin or irradiation were demonstrated *in vitro* for each NPC model. When using xenografts, Abexinostat by itself (12.5 mg/kg, BID, 4 days a week for 3 weeks) had significant anti-tumor effects against C17. Cooperative effects with cis-platin (2 mg/kg, IP, at days 3, 10 and 17) and irradiation (1Gy) were observed for the C15 and C17 xenografts. Simultaneously two types of biological alterations were induced in the tumor tissue, especially in the C17 model: a depletion of the DNA-repair protein RAD51 and a stronger *in situ* detection of the small viral RNA EBER1. Overall, these results support implementation of phase I/II clinical trials of Abexinostat for the treatment of NPC. A depletion of RAD51 is likely to contribute to the cooperation of Abexinostat with DNA damaging agents. Reduction of RAD51 combined to enhanced detection of EBER 1 might be helpful for early assessment of tumor response.

## Introduction

Nasopharyngeal carcinoma (NPC) is a malignant tumor arising from the epithelial lining of the nasopharynx. NPC represent a major public health problem worldwide [1]. In order of frequency, it is the third leading cause of virus-related human malignancy, ranking just behind hepatocellular carcinoma linked to HBV and HCV and cervix carcinoma associated with HPV. Incidence of NPC is particularly high in South China, especially in the Guangdong province (approximately 25 cases per 100 000 persons per year). In addition, there are areas of intermediate incidence whose extension has long been underestimated. These areas include much of Southeast Asia (Philippines, Indonesia, Thailand and Vietnam) and North Africa. Regardless of patient geographical origin, NPCs are constantly associated with the Epstein Barr Virus (EBV) (except for a very small number of highly differentiated atypical forms related to tobacco and alcohol which are observed in Europe and North America) [2]. No viral particles are detected in the tumor but the EBV genome is present in the nucleus of all malignant cells, encoding for a number of latent gene products, particularly non-translated RNAs (EBERs) and nuclear (EBNA1) or membrane proteins (LMP1 and LMP2). NPC is clearly a multifactorial disease, non-viral risk factors are germline genetic susceptibility and diet carcinogens which probably account for multiple acquired cellular genetic and epigenetic alterations [1].

The 5-year overall survival (OS) range from 60 to 95% for localized disease depending on the stage, while median OS is 24 months in case of metastatic disease [3], [4]. On average, NPCs are more radiosensitive and chemosensitive than other head and neck tumors and radiotherapy is the cornerstone of curative treatments. However, they still raise serious therapeutic concerns [5].

In the curative setting, because NPCs are often diagnosed at an advanced stage, the challenge is to reduce the rate of local and distant failures while reducing toxicities associated with radiotherapy. These aims have been partially met by advances in radiotherapy techniques and combination of radiotherapy with systemic treatments. The advent of Intensity Modulated Radiotherapy and 3-dimensional conformational radiotherapy now allow to limit the dose delivered to at-risk organs, and have been shown to lower the risk of xerostomia [6]. However, although xerostomia and other functional sequelae have become less frequent they have not been completely eliminated. Concomitant cis-platin based chemo-radiotherapy has proven to improve the disease-free and overall survival. Nevertheless, metastatic relapses and even locoregional failures still occur.

In the palliative setting, although NPCs are initially highly sensitive to chemotherapy, they often escape from treatment control after a few months and treatment options remain poor. Currently the main agents are cis-platin (CDDP), taxanes, gemcitabine and 5FU. Despite promising results obtained through *in vitro* studies, the use of targeted therapeutic agents is still limited. Additional progress will require the diversification of therapeutic agents available for systemic treatments. High expression of Epidermal Growth Factor Receptor (EGFR) and Vascular Endothelial Growth Factor (VEGF) in NPC has supported the rationale for clinical trials involving EGFR inhibitors (Cetuximab, gefitinib, and erlotinib) and VEGF inhibitors (bevacizumab, sunitinib; pazopanib)[7], [8], [9]. There have been several phase II trials using these compounds either alone or in combination with conventional treatment, with variable results and sometimes at the cost of severe toxicities (for instance hemorrhagic events with sunitinib)[10], [11], [12], [13]. To date, to our knowledge, there is no phase III trial of molecular targeted therapy in NPC.

Previous laboratory studies have reported a significant anti-tumor effect of a histone-deacetylase (HDAC) inhibitor Vorinostat using the C666-1 NPC cell line as the main experimental target [14]. The aim of our study was to use several NPC models, including patient-derived xenografts, to explore the therapeutic potential of a novel pan-HDAC inhibitor, Abexinostat. This compound is currently in Phase I/II clinical trials and being developed for the treatment of solid tumors as well as hematologic malignancies; the dose-limiting toxicity is the observation of thrombocytopenia [15], . One important aim of our preclinical study was to investigate cooperative anti-tumor effects of Abexinostat combined with either CDDP or radiotherapy. We here report that such cooperative anti-tumor effects were found in several of our models especially the two patient-derived xenografts (PDX) C15 and C17. Two types of biological alterations induced by Abexinostat were observed in these PDX: a decrease in the concentration of the DNA-repair protein RAD51 and enhanced *in situ* detection of the viral RNA EBER 1.

## Materials And Methods

### Ethics statement

The C15 and C17-PDX (patient-derived xenografts) tumor lines were derived from biopsies of patients admitted at the Institut Gustave Roussy. Some fragments of these biopsies which were in excess of what was required for diagnostic procedures were used to make xenografts. This was done with the approval of the multidisciplinary “Gustave Roussy NPC study group”, a structure gathering all leaders and several members of clinical departments/services and research laboratories involved in NPC treatment and research along with experts from other institutions. At that time, there was no ethics committee or IRB in Gustave Roussy. However the “Gustave Roussy NPC study group” was regarded as competent for medical, scientific and ethical matters. Members were meeting bi-monthly. Oral informed consent was given by the patients - and their parents for minor patients - to the Head of the ENT surgery service. According to the laws in force at that time (1983 to 1987), oral informed consent was sufficient for this procedure. Participant consent was recorded in the bi-monthly report of the “Gustave Roussy NPC study group”. The C15 and C17-PDX tumor lines have been initially described in 1988 [17]. Since this publication, they have been used in several studies (for example Sbih-Lammali et al., Cancer Research 1999; Klibi et al., Blood, 2009) [18], [19].

Procedures for nude mouse experiments were approved by the animal experimentation ethics committee of the Institut Gustave Roussy (CEEA-26 - decision # 2012-079).

### Xenografts and cell lines

We used a unique set of NPC models, including two EBV-negative cell lines (CNE1 and HONE1), one EBV-positive cell line (C666-1), and two patient-derived NPC EBV-positive xenografts (C15, C17) [20].

The C666-1 cells can be propagated *in vitro* as well as under the form of culture-derived xenografted tumors, while retaining the EBV genome [21], [22]. *In vitro*, C666-1 cells were grown in plastic flasks coated with collagen I (Biocoat; Becton-Dickinson, Franklin Lakes, NJ) using RPMI 1640 medium (Gibco-Invitrogen, Carlsbad, CA) supplemented with 25 mM HEPES and 7.5% foetal calf serum (FCS).

The C15- and C17-PDX (patient-derived xenografts) tumor lines are also EBV-positive. In brief C15 was derived from a primary tumor biopsy taken prior to any treatment from a 13-year old female patient from Morocco with a T4N3M0 NPC. She was initially treated with 3 cycles of COPAD (cyclophosphamide, vincristine, prednisolone, doxorubicin) resulting in 70% regression of the primary tumor and cervical lymph nodes. This neo-adjuvant chemotherapy was completed by external radiotherapy. However, bone metastases became symptomatic and visible on the CT scan immediately after the completion of radiotherapy. They were not stopped despite six cycles of vincristine, cyclophosphamide and CDDP. The patient died one year after her first admission. C17 was derived from the biopsy of a cutaneous metastasis taken from a multi-treated 38 year old male patient from France with a T3N3M0 NPC. The primary tumor and cervical lymph nodes were initially treated by radiotherapy. Multiple lung and skin metastases were discovered one year later. Complete remission was achieved after 6 cycles of chemotherapy combining CDDP, doxorubicin, bleomycin, vindesine and cyclophosphamide (six cycles). However two months later, lung and skin metastatic lesions were reactivated and found to be resistant to chemotherapy combining CDDP, bleomycin and 5-FU (4 cycles). The C17 xenograft was derived from a specimen obtained at this last stage of the disease.

The C15- and C17-PDX propagated by serial passages into nude mice were used between passages 250 and 270 and 230 and 250 respectively. For short-term *in vitro* experiments, single-cell suspensions were derived from C15 and C17 tumor fragments by collagenase cell dispersion as previously reported [23]. These cell preparations were designated C15 and C17 cells *ex vivo*.

The p53 status is known for all these NPC models. CNE1 and HONE 1 have a G to C transversion at codon 280 [24], [25]. C15 and C666-1 have retained wild-type on both alleles whereas C17 has a deletion of both alleles of the p53 gene [26].

### Pharmacological reagents

Abexinostat (Code: S78454-1; also coded PCI-24781-HCl by Pharmacyclics®) was provided by Technologie Servier (Orléans, France). For *in vitro* experiments, Abexinostat was solubilized at 10^−2^ M in DMS0, aliquoted and stored at −20°C. The frozen stock was thawed only once. Intermediate and final dilutions were prepared extemporaneously in complete culture medium protected from intensive light. For *in vivo* experiments, Abexinostat was diluted every two days in a sterile aqueous solution of 200 mM (2-hydroxypropyl)-beta-cyclodextrin (HPCD) protected from light. For example, to treat 25 mice weighing 25 g with a dose of 25 mg/kg for 2 days, 32 mg of compound were diluted in 20 ml of HPCD.

Cis-diammineplatinum (II) dichloride, named CDDP hereafter, was purchased from Sigma Aldrich (St. Quentin Fallavier, France). For *in vitro* experiments, CDDP was solubilized at 5 mM in DMS0, aliquoted and stored at −80°C. For *in vivo* experiments, CDDP was diluted in a 200 mM (2-hydroxypropyl)-beta-cyclodextrin (HPCD) sterile aqueous solution, aliquoted and stored at −20°C. For studies combining Abexinostat with CDDP, cell viability was assessed after concomitant treatment. For studies combining Abexinostat with irradiation, cells were pretreated with Abexinostat for at least 24 h prior to delivery of irradiation which was performed using an IBL 637 Cesium irradiator (Cis Bio International, Gif/Yvette, France).

### Cell viability assays

Cell viability was determined in short-term assays based on the reduction of MTT (CNE1, HONE1) or WST (a soluble form of MTT for C666-1, C15 and C17 *ex vivo*). MTT and WST were purchased from Sigma Aldrich. For these assays, cells were seeded in 96-well plates directly on the plastic at a density of 2×10^3^ (CNE1, HONE1), 3×10^4^ (C666-1) or 6×10^4^ (C15 and C17 *ex vivo*) cells per well using triplicates for each experimental conditions. Abexinostat and/or CDDP were added 1 to 4 hours after NPC cell plating. Overall, the total duration of cell incubation with Abexinostat and/or CDDP was about 48 h prior to MTT or WST assays. For studies combining Abexinostat with irradiation, Abexinostat treatment started on D1 with irradiation delivered 24 hours later (D2). The MTT/WST reaction was performed at least 24 hours post-irradiation. The calculation of the percentage of inhibition was based on the difference of OD between treated and untreated cells, after subtraction of the optical background.

Alternatively, *in vitro* growth assays were performed at low cell density to evaluate the effect of Abexinostat and combinations on the clonogenic potential of the most robust NPC cell lines (HONE1, CNE1 and C666-1). Assays were performed in six-well plates. HONE1 and CNE1 were seeded directly on the plastic at 2.5×10^2^ cells per well. C666-1 cells were seeded at 5×10^3^ cells per well on a feeder of irradiated normal human dermal fibroblasts (NHDF; Promocell, Heidelberg, Germany). Treatment with Abexinostat and/or CDDP was started 24 hours following cell plating. For experiments dealing with the combination of Abexinostat with radiotherapy, irradiation was performed 48 h after plating (i.e. 24 h after starting Abexinostat for cells treated by both agents). Treated and control cells were maintained in culture for time intervals allowing colonies of control cells to reach the adequate size for staining and counting. On average, 3 weeks were required for sufficient growth of the colonies. Two thirds of the culture medium was changed each 10 days without novel addition of the pharmacological compounds. At the completion of the incubation time, cell colonies were stained with a solution of Crystal Violet (Sigma Aldrich) in methanol (HONE1, CNE1) or a solution of Rhodanile Blue (Sigma Aldrich) in ethanol (C666-1) which is more specific for epithelial cells. Dried plates were then scanned and digitized to allow optical magnification and precise counting of cell colonies.

We used the Bliss additivism model to quantify the level of synergy in these drug-combination experiments [27]. The predicted Bliss additive effect C of two single compounds with effects A and B is: C = A+B−A·B (where A, B and C are percentages of inhibition). The excess over Bliss additivism (EOBA) was calculated by subtracting the predicted Bliss additive effect from the experimentally observed inhibition; it represents the excess of inhibition over the predicted response when 2 compounds are used in combination. Values greater than 10 are indicative of a synergy between the 2 compounds.

### Cell Protein Extraction and Western Blot Analysis

Proteins from cultured cells were extracted after 48 hours of treatment by lysis in RIPA buffer (50 mM Tris, 150 mM NaCl, 5 mM EDTA, 0.5% sodium deoxycholic acid, 0.5% NP-40, 0.1% SDS) supplemented with a protease inhibitor cocktail (Complete; Roche Molecular, Neuilly sur Seine, France). They were separated by SDS-PAGE and transferred to PolyVinyliDene diFluoride membranes (Immobilon, Millipore, Billerica, CA) by electroblot at 4°C for 90 minutes at 90 V or overnight at 45 V. Primary antibodies were mouse monoclonal antibodies directed against acetylated α-Tubulin (6-11B-1; Santa Cruz Biotechnology, Santa Cruz, CA, USA), PARP 1 (C-2-10; Calbiochem, San Diego, CA), β-actin and α-Tubulin (AC-74 and B-5-1-2, respectively; Sigma), rabbit polyclonal antibodies directed against RAD51 and RAD23B (sc-8349 and sc-67226, respectively; Santa Cruz Biotechnology). Blotted membranes were incubated with a secondary peroxidase-conjugated antibody, and chemiluminescent detection was done using the Immobilon Western Chemiluminescent HRP Substrate (Millipore, Billerica, CA).

### Nude mice experiments

Procedures for nude mouse experiments were approved by the animal experimentation ethics committee of the Institut Gustave Roussy (CEEA-26 - decision # 2012-079). Female swiss nude mice (nu/nu) at 6-8 weeks of age were xenografted by subcutaneous insertion of tumor fragments (average diameter of fragments 3 mm). The amount of grafted fragments was weighted for each mouse to ensure as much as possible equal tumor load in all treated and control mice (350 mg of wet fragments per mouse). Treatments started 10 to 15 days later depending on the type of xenograft (average volume of 100 mm^3^ at this stage). Groups of 10 mice were used for each therapeutic condition (vehicle−Abexinostat−vehicle+other modality−Abexinostat + other modality). Abexinostat was given from D1 to D4, D8 to D11 and D15 to D17 (intra-peritoneal (IP) injections of 12.5 mg/kg twice a day, with an interval of 6 to 8 hours). CDDP was given IP at a dose of 2 mg/kg at D3, D10 and D17. Treatment by external irradiation was done at D3, D10 and D17. Selective tumor irradiation was delivered using a Varian NDI 226 X-ray tube (0.87 Gy/mn) under a tension of 200 KV at 15 mA, with a 0.2 mm cooper filter and a skin-source distance of 21 cm. Mice were sacrificed at day 18 following the onset of the treatment. Caliper tumor measurements and weighing of the animals were done at least three days a week. Animals with weight losses greater than 20% were sacrificed. Drug doses were adapted to weigh losses of less than 20%. In all cases, animals were sacrificed at day 18 following the onset of the treatment. For biochemical assays performed on tumor material treated *in vivo*, xenografted mice were treated for 48 hours prior to animal sacrifice and tumor collection.

### Statistical analysis of xenografted tumor growth

The statistical analysis was done on Log [tumor weight (mg) +1]. The synergy was analyzed by a two-way [Abexinostat x PI (proposed interactor)(CDDP or irradiation)] analysis of variance. When the (Abexinostat x PI) interaction was significant, complementary analyses of Abexinostat effect was done at fixed levels of PI. Otherwise a profile analysis was performed: REML method and Satterthwaite degrees of freedom were used. Threshold: 10% for interaction term, 5% for principal effects.

When the (Treatment x Time) interaction was significant, complementary analyses of Treatment effect was done at fixed levels of Time, otherwise an analysis of all time points taken together was performed. Each two-by-two comparison was performed, using a Tukey correction for multiplicity (REML method, Satterthwaite degrees of freedom, ARH variance-covariance matrix). Threshold: 10% for interaction term, 5% for principal effects.

### RNA extraction and transcript assessment by real-time RT-PCR

Total RNA from cultured cells and xenografted tumors was extracted using the mirVana isolation kit (Life Technologies, Saint-Aubin, France) skipping the procedure for separation of small RNAs. Real time RT-PCR was done using the “TaqMan RNA to C_T_ 1-Step” kit from Life Technologies where RNA is processed in the same tube for reverse transcription and subsequent PCR amplification of one specific RNA target template. In each tube, the same oligo-nucleotide is used as an RT primer and a PCR anti-sense primer, successively. For each RNA target, RT-PCR reactions were done in duplicate or triplicate. The TaqMan primers/probe oligonucleotide mixes were from Life Technologies. For RAD51, GAPDH and TFRC1 we used standard oligonucleotide sets, Hs00153418, Hs03929097 and Hs00174609, respectively. For EBER1 we used custom made oligonucleotides and internal probe (“custom gene expression Taqman assay”; forward primer: 5′-GGACCTACGCTGCCCTAGA-3′, reverse primer: 5′- GGGACGGGTGGCTACAG-3′; internal probe: 5′-CCTCCCTAGCAAAACC-3′). For one single reaction, the following reagents were mixed: 0.31 µl RT enzyme mix (containing Arrayscript reverse transcriptase), 6.25 µl PCR mix (containing Ampli Taq Gold DNA polymerase) and 0.63 µl primers/probe mix (final concentration of the primers and FAM-labeled probe: 0.9 and 0.25 µM respectively), 5.31 µl RNA solution (containing 5 or 20 ng of RNA) for a total reaction volume of 12.5 µl. Reaction were run in a StepOnePlus thermal cycler (Life Technologies) beginning by one step at 45°C for 30 min (RT reaction) and one step at 95°C for 10 min (release of single strand DNA and activation of the DNA polymerase). These initial steps were followed by 40 cycles including one step at 95°C for 15 sec and one step at 60°C for one min. Results were analyzed using the StepOne software.

### EBER1 *in situ* hybridization

Pieces of C666-1 and C17 xenografts were fixed in 4% paraformaldehyde, paraffin-embedded and cut in 4 µM sections. EBER detection was performed by *in situ* hybridization using the INFORM EBER Probe (specific of the EBER1 RNA) and the i-VIEW Blue Detection Kit from Ventana-Roche (Ref 800–2842 and 800-092 respectively)(Tucson, AZ, USA).

## Results

### Abexinostat exerts significant cytotoxic effects on EBV-positive NPC cells treated *in vitro*

The cytotoxicity of Abexinostat on NPC cells was investigated using target cells from EBV-negative (HONE1, CNE1) and EBV-positive (C666-1, C15 and C17) NPC models. The human diploid lung fibroblasts MRC5 were used as a non-malignant reference [28]. Cells were treated with various concentrations of the pharmaceutical agents during 48 hours. Dose-response curves were determined using short-term assays based on high density cultures and final measurements by reduction of MTT (HONE1 and CNE1) or WST (C666-1, C17 and C15). As shown in Table 1, MRC5 was relatively resistant to Abexinostat (IC 50 of 500 nM). The IC50 for the EBV-negative NPC models was in the same range. In contrast, the EBV-positive NPC models were much more sensitive, especially C17 (IC50 = 150 nM). The cytotoxicity of CDDP was investigated in parallel experiments resulting in IC50 values about 10-fold higher, in the range of 3 to 10 µM. In contrast with our observations about Abexinostat, the EBV-positive NPC cells were somehow more resistant to CDDP than the 2 EBV-negative cell-lines. Clonogenic assays for Abexinostat were performed for three cell lines able to sustain prolonged growth at low density, HONE1, CNE1 and C666-1. For the EBV-negative HONE1 cell-line, values of IC50 derived from the clonogenic assays were identical to those obtained using high density cell cultures. In contrast, they were reduced for CNE1 (EBV-negative). A greater reduction was recorded for C666-1 (EBV-positive), from 220 (high density culture) to 70 nM (clonogenic assay) (Table 1). This suggests that homologous cellular interactions have a special role in the resistance of C666-1 cells to Abexinostat.

**Table 1:** Sensitivity of NPC cells treated *in vitro* with Abexinostat or CDDP used as single agents.

Cell type	Abexinostat IC50		CDDP IC50	
	MTT/WST	Clonogenic assay	MTT/WST	Clonogenic assay
**MRC5**	500 nM		15 µM	
**HONE1**	400 nM	400 nM	3 µM	1.8 µM
**CNE1**	700 nM	300 nM	3 µM	1.8 µM
**C666-1**	220 nM	70 nM	10 µM	4 µM
**C15**	200 nM		5 µM	
**C17**	150 nM		8 µM	

### Cooperative cytotoxic effects of Abexinostat combined with CDDP or irradiation on NPC cells treated *in vitro*

**Figure 1:**
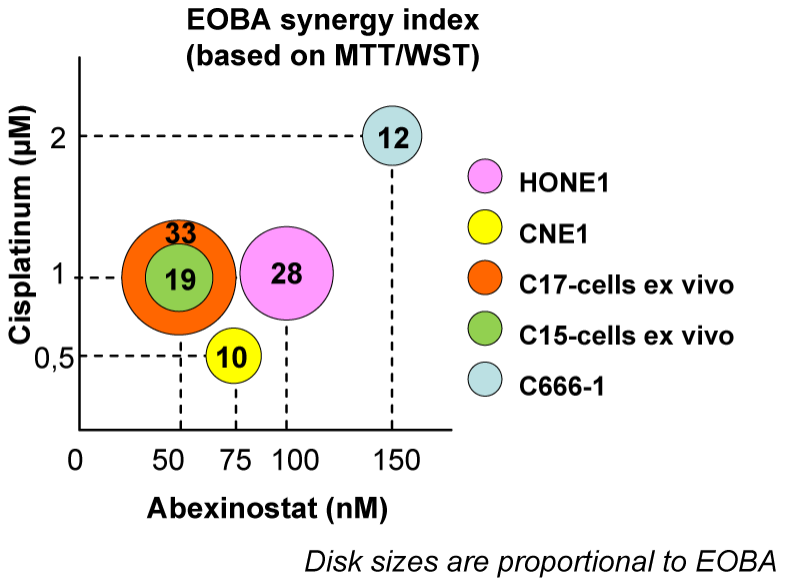
Short term culture assays on NPC cells treated *in vitro* with Abexinostat plus CDDP. The best combinations of drug dosages are represented graphically. EBV-negative (HONE1 and CNE1) and EBV–positive NPC cells (C15 cells *ex vivo*, C17 cells *ex vivo* and C666-1 cells) were treated for 48 h with combinations involving various concentrations of Abexinostat (25, 50, 75 and 150 nM) and CDDP (0.5, 1 and 2 µM). Cell viability was determined in short term assays based on the reduction of MTT (CNE1, HONE1) or WST (C15 *ex vivo*, C17 *ex vivo* and C666-1) using triplicates for each experimental conditions. For each NPC model, the result of the most successful combination of drug dosages is depicted as a color disk whose size is proportional to the EOBA index. This index mentioned as a two-digit number reflects the level of synergy for the combined treatment: values greater than 10 are indicative of a synergy between the 2 compounds (see the Materials and Methods section for additional explanations). The X and Y coordinates of the disk stand for the corresponding concentrations of Abexinostat and CDDP respectively. For CNE1 and C666-1, the effects of the combined treatment were only additive. The highest levels of synergy were recorded for C17-cells *ex vivo*, HONE1 and C15-cells *ex vivo*.

**Figure 2:**
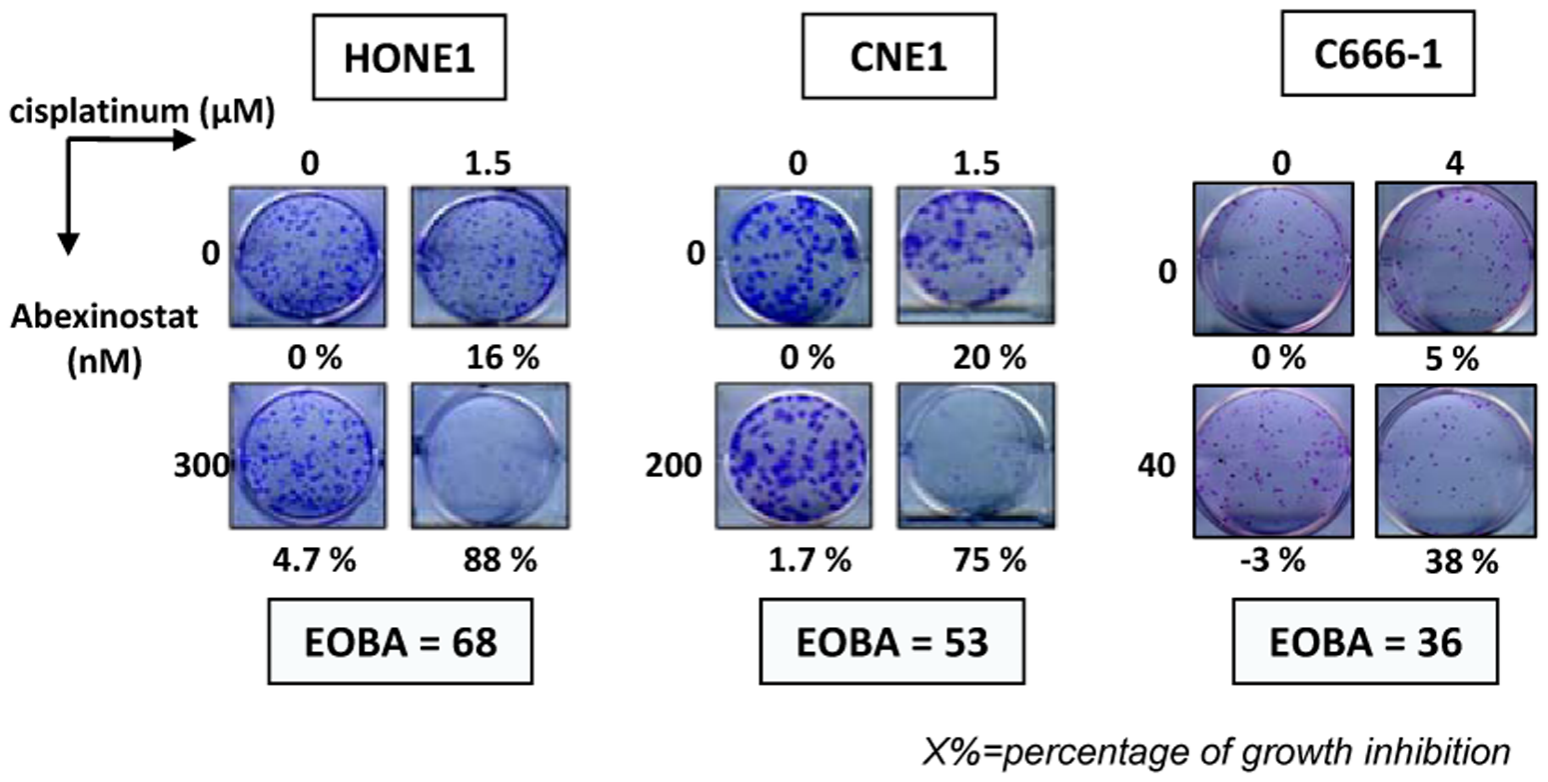
Clonogenic assays on NPC cells treated *in vitro* with Abexinostat combined with CDDP. The experiments corresponding to the most successful combinations of drug dosages are presented for HONE1, CNE1 and C666-1. Cells were plated as explained in the Materials and Methods section. Simultaneous treatment by Abexinostat and CDDP was started 24 hours following cell plating. About 3 weeks later, cell clones were numbered for calculation of the percentages of growth inhibition (which are mentioned under the picture of each well). For C666-1 cells treated with Abexinostat by itself (40 nM), clonal growth was slightly enhanced instead of being inhibited. The “excess over Bliss additivism” (EOBA) which reflects the level of synergy for the combined treatment is mentioned for each NPC model (see the Materials and Methods section for the explanation of this index). Values greater than 10 are indicative of a synergy between the 2 compounds. The panel of each NPC model is representative of 3 similar experiments.

CDDP is a major standard therapeutic agent for the treatment of nasopharyngeal carcinomas. Therefore, our next aim was to investigate a possible cooperative effect of Abexinostat and CDDP against NPC cells treated *in vitro*. NPC cells were treated for 48 h with low concentrations of Abexinostat or/and CDDP These concentrations were in the range of IC50/8 to IC50/2, in other words, 25 to 150 nM (Abexinostat) and 0.5 to 2 µM (CDDP). The impact of single or combined agents was first measured by MTT or WST assays. Levels of cooperation between the 2 agents were assessed using the Bliss additivism model [27]. Results obtained with the most successful combinations of drug dosages are displayed in Figure 1. The combination of the 2 compounds was qualified as synergistic when the excess over Bliss additivism (EOBA) was above a threshold of 10. The best synergies were recorded for C17 and to a lesser extent HONE1 and C15 (Figure 1). In contrast, there was almost no cooperation of the two agents when treating C666-1 and CNE1. For HONE1, CNE1 and C666-1, the cooperation between Abexinostat and CDDP was further investigated using clonogenic assays. Results obtained with the most successful combinations of drug dosages are displayed in Figure 2. Synergistic effects greater than those observed with MTT/WST assays were recorded for HONE1 and C666-1. A cooperative effect which was not apparent when using the MTT assay was also demonstrated for CNE1.

**Figure 3:**
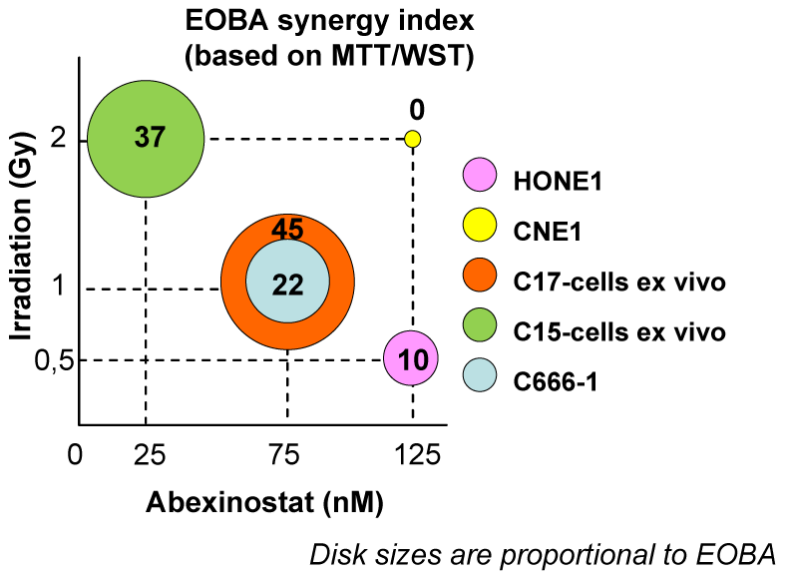
Short term culture assays on NPC cells treated *in vitro* with Abexinostat plus external irradiation. The best combinations of treatment dosages are represented graphically. EBV-negative (HONE1 and CNE1) and EBV–positive NPC cells (C15 cells *ex vivo*, C17 cells *ex vivo* and C666-1 cells) were treated for 48 hours with combinations involving various concentrations of Abexinostat (25, 50, 75 and 150 nM) and doses of irradiation (1, 2 and 4 Gy 24 h after cell plating). Cell viability was determined in short term assays based on the reduction of MTT (CNE1, HONE1) or WST (C15 *ex vivo*, C17 *ex vivo* and C666-1). For each NPC model, the result obtained with the most successful combination of treatment dosages is depicted as a color disk whose size is proportional to the EOBA index (mentioned as a two-digit number). The X and Y coordinates of the disk stand for the corresponding concentration of Abexinostat and dose of irradiation, respectively. Note that for HONE1 and CNE1, the effects of the combined treatment were only additive. The highest levels of synergy were recorded for C15 and C17 cells *ex vivo*.

**Figure 4:**
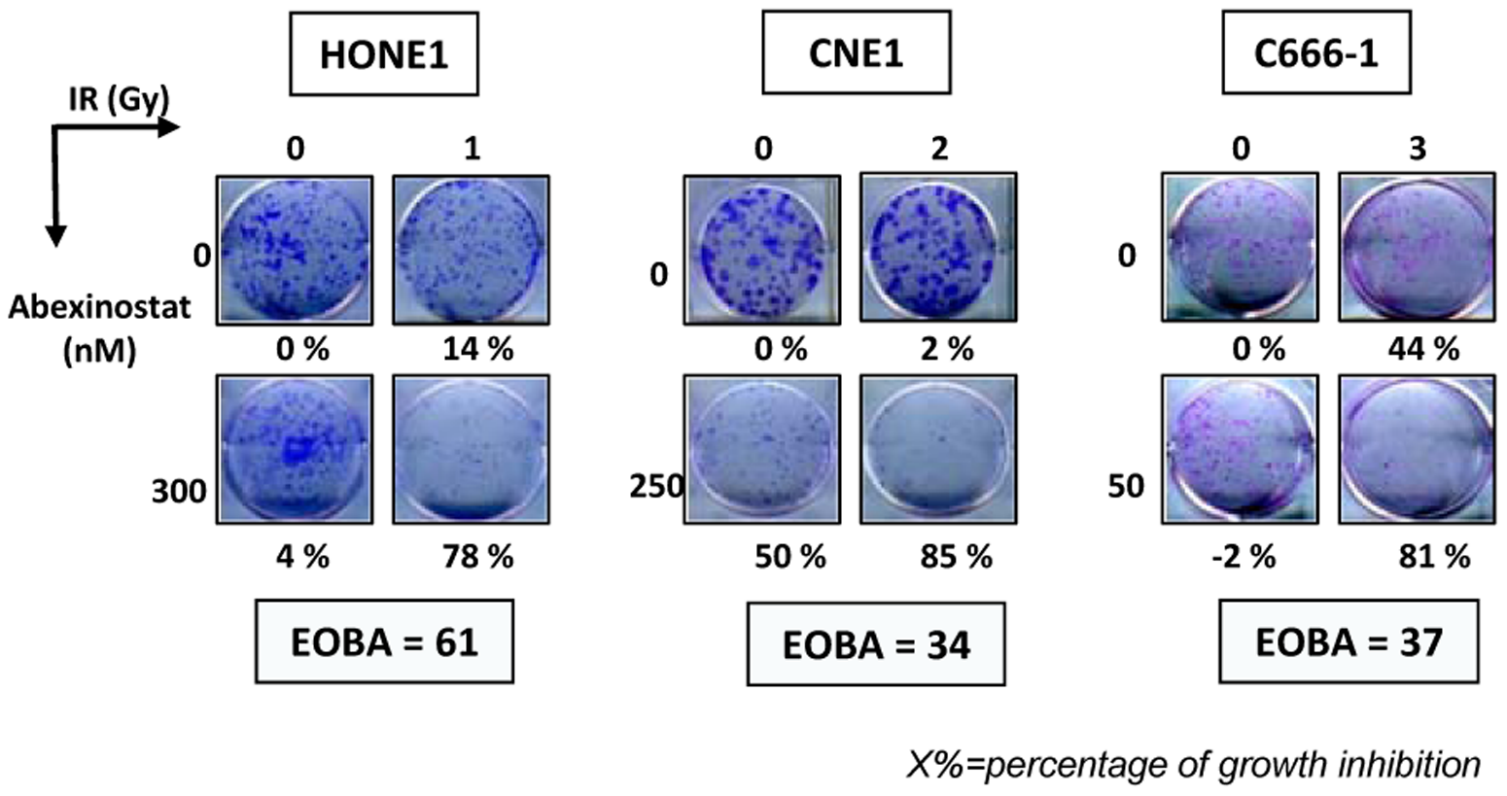
Clonogenic assays on NPC cells treated *in vitro* with Abexinostat combined with external irradiation. The experiments corresponding to the most successful combinations of treatment dosages are presented for HONE1, CNE1 and C666-1. Cells were plated as explained in the Materials and Methods section. Treatment by Abexinostat was started 24 hours following cell plating and irradiation was done 24 h after the onset of Abexinostat. About 3 weeks later cell clones were numbered for calculation of the percentages of growth inhibition (which are mentioned under the picture of each well). For C666-1 cells treated with Abexinostat by itself (50 nM), clonal growth was slightly enhanced instead of being inhibited. The EOBA which reflects the level of synergy for the combined treatment is mentioned for each NPC model. The panel of each model is representative of 3 similar experiments.

External irradiation is another major therapeutic arm for the treatment of nasopharyngeal carcinomas. Therefore, we investigated the potential synergistic effects of Abexinostat combined with external irradiation. Abexinostat was applied for 48 h at concentrations in the range of IC50/8 to IC50/2, in other words, 25 to 150 nM. Irradiation was delivered 24 h after the onset of Abexinostat at doses of 1, 2 or 4 Gy. The impact of single or combined agents was first measured by MTT or WST assays. Results obtained with the most successful combinations of treatment dosages are displayed in Figure 3. The best cooperative effects were obtained for C15 and C1*7*. When using high density cultures only minimal cooperation was recorded for C666-1 and no synergy was observed for the EBV-negative cell lines, HONE1 and CNE1. However, synergistic effects were demonstrated for HONE1, CNE1 and C666-1 cells when making clonogenic assays (Figure 4).

In summary, C17 cells were highly sensitive to both modalities of combined treatments. On the other hand, C15 cells were highly sensitive to the combination of Abexinostat with irradiation but only mildly sensitive to the combination of Abexinostat with CDDP. For HONE-1, CNE1 and C666-1 a robust synergy was observed only when using clonogenic assays.

### Cooperative tumor growth reduction of NPC xenografts treated with Abexinostat combined with CDDP or external irradiation

On the basis of preliminary experiments, we adopted the following regimen for administration of Abexinostat to nude mice bearing xenograted tumors: 12.5 mg/kg IP BID 4 days a week for 3 weeks. With regards to CDDP, preliminary experiments were done on all three types of NPC xenografts to evaluate the magnitude of tumor growth reduction under various doses (2, 4 and 8 mg/kg, once a week for 3 weeks) (Fig S1, S2 and S3). At the dose of 2 mg/kg, no growth reduction was observed; on the contrary, we consistently noted a small increase in tumor growth under treatment for all three xenografts. At 4 mg/kg, the rate of tumor growth was reduced but not to the same extent in C17 (2.8 fold reduction at day 18 post-treatment), C15 (1.7 fold) and C666-1 (1.3 fold). At 8 mg/kg – the maximum tolerated dose – tumor growth was virtually blocked for C15 and C17 and only mildly reduced for C666-1. In summary, the C15 and C17 xenografts were sensitive to CDDP whereas C666-1 was poorly sensitive.

**Figure 5:**
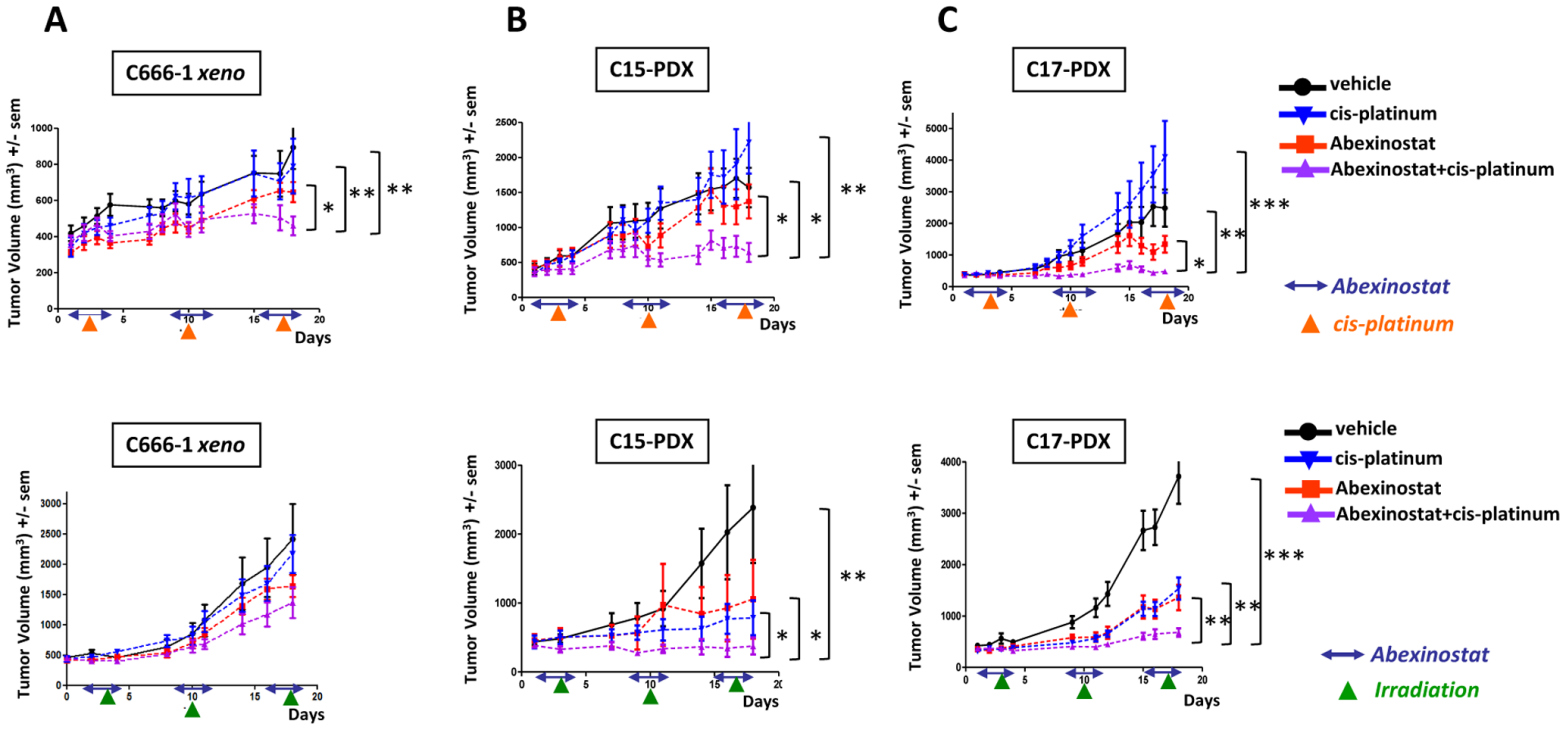
Cooperative cytotoxic effects of Abexinostat combined with CDDP or external irradiation on xenografted NPC tumors. Treatment was started 12 days following subcutaneous graft of tumor fragments (350 mg per mouse). Groups of 10 mice were used for each therapeutic condition (vehicle−Abexinostat−vehicle + CDDP or irradiation−Abexinostat + CDDP or irradiation). Abexinostat was given from D1 to D4, D8 to D11 and D15 to D17 (two intra-peritoneal (IP) injections of 12.5 mg/kg per day with an interval of 6 to 8 hours). CDDP was given IP at a dose of 2 mg/kg at D3, D10 and D17 (upper panel). Selective irradiation of the tumor volume was done at D3, D10 and D17 (lower panel). Caliper tumor measurements and weighing of the animals were done at least 3 days a week. Mice were sacrificed at day 18 following the onset of the treatment. Error bars are based on SEM. **A - Cooperative tumor growth reduction of C666-1 NPC xenografts when treated with Abexinostat combined with CDDP but not with irradiation.** Tumor growth reduction is statistically significant at day 18 for the combination of Abexinostat with CDDP by comparison with tumors treated with the vehicle, Abexinostat alone or CDDP alone (upper graph). The combination of Abexinostat with irradiation has no statistically significant effects using this NPC model (lower graph). **B - Cooperative tumor growth reduction of C15 NPC xenografts treated with Abexinostat combined with CDDP or irradiation.** Tumor growth reductions are statistically significant for the combinations of Abexinostat with CDDP (upper graph) or with irradiation (lower graph) by comparison with tumors treated with the vehicle, Abexinostat, CDDP or irradiation used as single agents. **C - Cooperative tumor growth reduction of C17 NPC xenografts treated with Abexinostat combined with CDDP or irradiation.** Tumor growth reductions are statistically significant for the combinations of Abexinostat with CDDP (upper graph) or with irradiation (lower graph) by comparison with tumors treated with the vehicle, Abexinostat, CDDP or irradiation used as a single agent.

For the combined treatment with Abexinostat, we chose the lightest regimen: CDDP 2 mg/kg once a week for three weeks (days 3, 10 and 17) (Figure 5). The result was a dramatic tumor growth reduction for C17 and a still substantial reduction for C15 and to a lesser extent for C666-1 xenograft. In all cases, the combined treatment was more efficient than each drug used as a single agent.

For the combined treatment with radiotherapy, selective tumor irradiation was given at a dose of 1 Gy once a week for three weeks. This combination resulted in a major reduction of tumor growth for C15 and C17. In contrast, for C666-1 no benefit was demonstrated by statistical analysis.

In summary, the sensitivity to both modalities of combined treatment was high for C17, average for C15 and low or absent for the C666-1 xenograft.

### Protein modifications induced by Abexinostat in NPC cells treated *in vitro*

**Figure 6:**
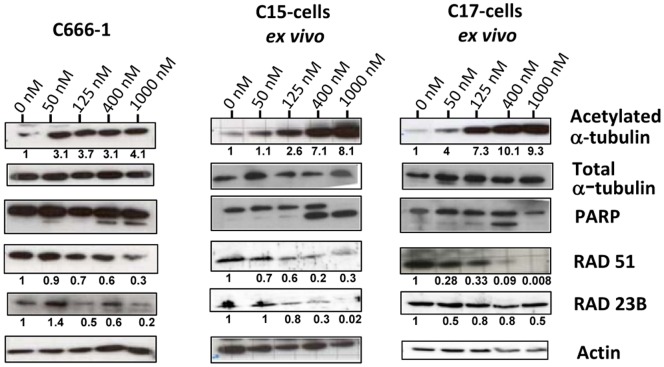
Protein modifications induced by Abexinostat in EBV-positive NPC cells treated *in vitro*. These experiments were done on C666-1 cells routinely propagated *in vitro* (C666-1) and on cells resulting from collagenase dispersion of C15 and C17 patient-derived xenografts (C15 and C17 cells *ex vivo*). Treatment with Abexinostat at various concentrations (0, 50, 125, 400 and 1000 nM) was applied continuously for two days (D1 and D2). Cell proteins were then extracted and separated on PAGE gels (20 µg/lane). Parallel Western blots were done for acetylated α-tubulin, α-tubulin, PARP1, RAD51, RAD23B and actin. Protein abundance in each lane was evaluated by densitometry. For loading normalization, all proteins were referred to actin except acetylated α-tubulin which was referred to total α-tubulin. Protein abundance in lanes corresponding to untreated samples was arbitrarily set at 1. Surprisingly PARP cleavage is observed for C17 cells treated with 400 nM but not 1000 nM Abexinostat. Because C17 cells were very sensitive to Abexinostat, we suspect that when treated with a high concentration of the compound they died by a non-apoptotic mechanism possibly related to necrosis.

Protein modifications induced by Abexinostat were investigated *in vitro* in order to better understand molecular mechanisms underlying the response of EBV-positive NPC cells. C666-1 cells (permanently propagated *in vitro*) and cells resulting from collagenase dispersion of C15- and C17-PDX were subjected to *in vitro* treatment by increasing doses of Abexinostat for 48 h. For all three models, an increase in α-tubulin acetylation was obvious for concentrations of Abexinostat above 100 nM (approximately IC50/2) (Figure 6). It was of larger magnitude for C15 and C17 cells with more obvious dose dependency by comparison with C666-1 cells. Higher concentrations of Abexinostat were required to observe a PARP cleavage indicative of caspase-dependent apoptosis (in the range of 400 nM, approximately IC50 X 2). We sought to detect a depletion of RAD51 in NPC cells treated with Abexinostat as previously reported for the human colonic carcinoma cells HCT116 and multiple myeloma cell lines treated by HDAC inhibitors [29], [30]. RAD51 protein concentrations were indeed decreased by Abexinostat in C666-1, C15 and C17 cells (Figure 6). However dose/response relationships were somehow variable depending on the model and even in a given model through various experiments (Figure 3 and 4 and data not shown). In summary, while in C15 and C17 cells RAD51 protein abundance was consistently reduced by at least 50% under Abexinostat at 200 nM for 48 h, its depletion was often less marked for C666-1 cells. There was also a trend toward a depletion of RAD23B under treatment by Abexinostat. In a report by Fotheringham et al. (2009), it has been shown that high amounts of pre-treatment RAD23B in myeloma and cutaneous T-cell lymphoma cells are predictive of a high sensitivity to HDAC inhibitors [31]. Therefore pre-treatment concentrations of RAD23B were compared in the C666-1, C15 and C17 models prior to any treatment (Figure S4). We found no relationships with tumor response to Abexinostat.

**Figure 7:**
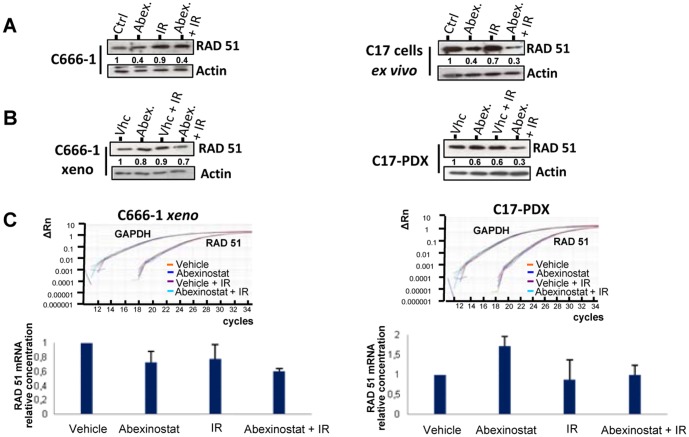
RAD51 protein and mRNA in NPC cells treated with Abexinostat combined with irradiation. A – RAD51 protein concentrations in NPC cells treated *in vitro.* Experiments were performed on cells resulting from collagenase dispersion of the C17 patient-derived xenograft (C17 cells *ex vivo*) and on C666-1 cells routinely grown *in vitro* (C666-1). Treatment with Abexinostat (200 nM in culture medium) was applied continuously for two days (D1 and D2). Irradiation was given early on D2. Protein extracts were prepared from untreated cells (Ctrl), cells treated with Abexinostat alone (Abex.), irradiation alone (1Gy) (IR) and combination of both (Abex.+IR). Protein extracts (20 µg/lane) were subjected to PAGE gel separation. Western blotting was performed for detection of RAD51 and actin. RAD51 protein abundance was evaluated by densitometry with normalization on the actin bands detected on the same blotted membrane. It was arbitrarily set at one in lanes corresponding to untreated samples (Ctrl). In C17 and C666-1 cells, RAD51 concentration was decreased to almost the same extent by Abexinostat or Abexinostat plus irradiation. These data are representative of three similar experiments. B – RAD51 protein concentrations in xenografted NPC cells treated *in vivo*. Mice bearing C17-PDX tumors or xenografted C666-1 cells (C666-1 xeno) were treated during two days (D1 and D2) with vehicle alone, Abexinostat (25 mg/kg BID), vehicle + selective tumor irradiation (2Gy early on D2) or Abexinostat + irradiation. Tumors were collected at D3 and split in fragments used either for protein or RNA extraction (see Figure 4C). Tumor protein extracts were subjected to Westen blot and densitometry as reported for *in vitro* cell extracts in the legend of Figure 4A. For the C17-PDX, we could observe a reduction in the tumor concentration of RAD51 under treatment by Abexinostat. This reduction was even more marked when Abexinostat was combined to irradiation. In contrast, the depletion of RAD51 was modest for C666-1 xeno. These data are representative of three similar experiments. C – RAD51 mRNA concentrations in xenografted NPC cells treated *in vivo*. Total RNA was extracted from fragments of xenografted tumors subjected to various treatments in order to investigate the abundance of the RAD51 messenger RNA by real time RT-PCR. Examples of amplification curves are given for RAD51 and GAPDH (glyceraldehyde-3-phosphate dehydrogenase) mRNA which was used as an internal reference. The relative concentrations of RAD51 mRNA obtained for each type of treatment were calculated by the 2^−ΔΔCt^ method. RNAs from the tumors treated with the vehicle only (vhc) were used as external references or calibrators. Values given in the histogram are means of two distinct experiments. The impact of Abexinostat on RAD51 mRNA was modest and not consistent for C666-1 and C17. For example, it was decreased in the C666-1 xenograft treated with Abexinostat only whereas it was mildly increased in the corresponding C17-PDX. In the C17-PDX treated with Abexinostat combined to irradiation the depletion of the RAD51 mRNA was modest in comparison with the protein depletion in the same tumor fragments (Figure 5B).

In the next step, we investigated RAD51 protein depletion in C666-1 and C17 cells treated *in vitro* with Abexinostat combined to irradiation. Irradiation was delivered to NPC cells 24 h after the onset of Abexinostat. The depletion of RAD51 was at least as intense under Abexinostat plus irradiation as under Abexinostat alone (Figure 7A). The same trend was observed when Abexinostat was combined to CDDP (see Figure S5). It was also observed in C666-1 and C17 xenografts treated *in vivo* with systemic injections of Abexinostat and selective tumor irradiation. However, to improve the consistency of RAD51 reductions, we had to increase the doses of Abexinostat and irradiation to 25 mg/kg BID and 2 Gy respectively. Nevertheless, through numerous experiments, regardless of the doses that were used, the decrease in the amount of RAD51 was more marked for C17-PDX (highly sensitive to Abexinostat plus radiotherapy in terms of growth reduction) than for C666-1 xenograft (not sensitive). The amount of RAD51 protein was reduced by 70 and 30%, respectively (Figure 7B). Although relatively modest, this difference was highly consistent. Next, we intended to determine whether RAD51 protein depletion was related to altered transcription as previously reported for HCT116 cells treated with Abexinostat [29]. The RAD51 messenger RNA was investigated by real time RT-PCR in C666-1 and C17 xenografts treated with Abexinostat, radiotherapy or the combination of both. Only mild variations were observed which were not consistent with observed RAD51 protein reduction (Figure 7C). For example, for the C17-PDX, the reduction of the mRNA was only 20% to be compared with a 70% reduction of the protein. This suggests that, to some extent, the amount of RAD51 protein was decreased independently of alterations in transcription for example by enhanced protein degradation as reported for myeloma cells treated with Vorinostat and irradiation [30].

### Modifications of *in situ* EBER 1 detection

**Figure 8:**
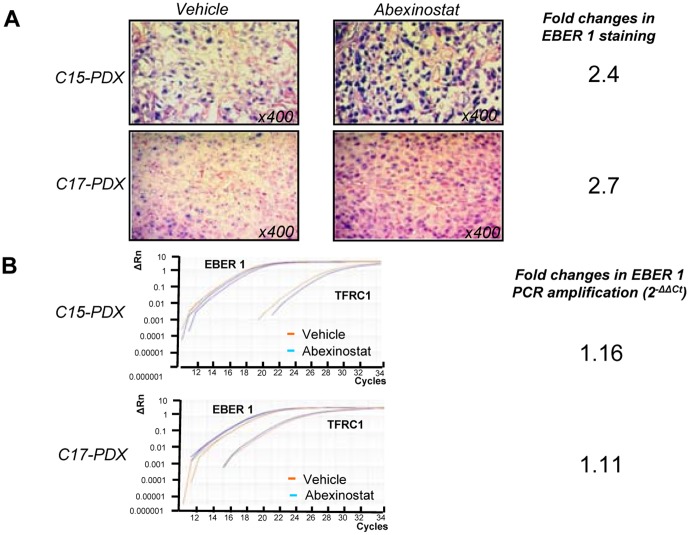
Detection of EBER1 in NPC patient-derived xenografts (PDX) treated with Abexinostat. Xenograted C15 and C17 tumors of mice treated for 2 days (D1 and D2) with Abexinostat (12.5 mg/kg BID) or vehicle were harvested early on D3. Fragments from the same tumors were split for detection of EBER1 either by *in situ* hybridization on tissue sections or tumor RNA extraction and real time RT-PCR. **A - Detection by** ***in situ*** **hybridization.** Quantification of staining was made using the Image J software following acquisition of data from tissue sections with the Axovision digital image processing software from Zeiss (Le Pecq, France). Regions of interest (ROI) with comparable cell densities were defined in sections from tumors treated with Abexinostat or the vehicle. A threshold of minimal staining was set at the same value for these two experimental conditions. The percentage of stained areas inside the ROIs was taken as a staining index. Using this parameter we found that treatment with Abexinostat resulted in a 2.4 and 2.7 fold increases in the amount of EBER staining in the C15 and C17 xenografts, respectively. **B - Detection by real time RT-PCR.** Examples of amplification curves are given for EBER1 and TFRC1. EBER1 RNA fold-changes were calculated by the 2^−ΔΔCt^ method using RNA from the tumors treated with the vehicle (vhc) as the external reference whereas the TFRC1 mRNA was used as the internal reference. On the basis of these data, the impact of Abexinostat on the total amount of EBER1 RNA contained in NPC xenografts was apparently marginal.

*In situ* hybridizations of the viral RNA EBER1 were done on tissue sections of NPC xenografts following treatment with the vehicle or Abexinostat in order to improve our description of its anti-tumor effects. We found a consistent increase in the intensity of EBER1 staining in xenografts treated with Abexinostat. This is depicted in Figure 8A for the C15 and C17 xenografts. Regarding C666-1 xenografts, the effect of Abexinostat on EBER1 staining was difficult to assess because staining is already quite intense in basal conditions. Finally, we investigated the amount of EBER1 RNA by tumor extraction of total RNA and real time RT-PCR (Fig 8B). By this procedure, we found no changes parallel to what was observed by *in situ* hybridization. This suggests that the increase in staining intensity on tissue sections does not result from a greater amount of target RNAs but from their better accessibility to the hybridizing probe.

## Discussion

Biological and pharmacological investigations of NPC cells have long been hampered by the lack of genuinely representative cell lines, especially cell lines permanently propagated *in vitro* and retaining the EBV genome. One unique feature of this study is that it has investigated the tumor suppressive effect of a novel HDAC inhibitor simultaneously on 5 NPC models including 2 patient-derived xenografts (PDX; C15 and C17). Because two classical EBV-negative NPC cell lines, HONE1 and CNE1, have been often used in previous publications, they were included in our study at the stage of *in vitro* investigations. Overall when combining high density cell culture assays and the more sensitive clonogenic assays we have found a cytotoxic effect of Abexinostat on all five NPC cell lines. However, cells from the C17-PDX were the most sensitive in most assays using either Abexinostat alone or in combination with CDDP or irradiation. Systemic treatments were performed on xenografted NPC tumors using C666-1 (culture-derived xenograft) as well as C15 and C17 (patient-derived xenografts). Again the C17 model was the most sensitive to treatment combining Abexinostat with CDDP or external irradiation whereas the C666-1 model was poorly sensitive or unsensitive.

With regard to NPC therapy in the palliative setting, it is noteworthy that the C17 xenograft was derived from a metastatic NPC which had acquired secondary resistance to CDDP. However our study shows that the corresponding PDX has retained sensitivity to high doses of CDDP as well as to low doses of CDDP combined to Abexinostat (Fig S1, S2, S3 and Fig 5). This suggests that in patients with secondary resistance to CDDP, it might be worth to use a combination of Abexinostat with relatively low doses of CDDP in order to overcome resistance with acceptable tolerance for patients often highly pretreated.

With regard to NPC therapy in the curative setting, the synergy with irradiation demonstrated for the C15- and C17-PDX is another very interesting finding. Indeed, one major challenge for upfront NPC therapy is to achieve treatment of the primary tumor and cervical metastases as well as of the distant micro-metastatic disease. This probably explains the better outcome achieved by concomitant chemoradiotherapy demonstrated by meta-analyses in endemic and non-endemic populations [32], [33]. Several groups are now moving forward to achieve even better prevention of distant metastasic relapses. They intend to add neoadjuvant or adjuvant chemotherapy prior or following chemoradiotherapy. Unfortunately, these groups often have to deal with unacceptable secondary effects that may impair the relative dose-intensity of treatment. For example, CDDP is often ill-tolerated when it is used for adjuvant chemotherapy consecutively to chemoradiotherapy [34]. In the light of our results, one can imagine using Abexinostat in combination with radiotherapy to save the benefit of CDDP for neoadjuvant/adjuvant treatment or maybe to attempt the opposite sequence. In concurrent chemoradiotherapy, systemic agents are expected to provide a radioensitizing effect in addition to a direct, general anti-tumor effect. Abexinostat appears to be able to fulfill both requirements, at least when dealing with NPCs that would behave like the C17-PDX.

The above-conclusion underlines the importance of the identification of markers monitoring the tumor response to Abexinostat. Publications from other groups have reported modifications of signaling pathways related to DNA-repair, cell cycle regulation or control of cell survival in various types of malignant cells treated with HDAC inhibitors, including the enhanced degradation of RAD51 and FLIP or the enhanced expression of CDKN1A [30], [35], [36]. In our investigation of NPC cells treated with Abexinostat, we have found no evidence of changes in the amount of FLIP or CDKN1A (data not shown). In contrast with Fotheringham et al. (2009) we did not observe a relationship with the pre-treatment level of RAD23B (Figure S4) [31]. Overall, the decrease in the cell concentration of RAD51 was the strongest and most consistent change (Figure 6 and data not shown). Moreover, our results suggest that Abexinostat can cooperate with irradiation (IR) in the degradation of RAD51. This might be explained in light of a report by Chen et al. [30]. They have shown that Vorinostat inhibits the chromatin binding of RAD51 induced by irradiation in myeloma cells and simultaneously blocks its accumulation in treated cells. One can speculate that Abexinostat alters the mobilization of RAD51 induced by irradiation and blocks its accumulation or even favors its degradation.

The RAD51 protein is a key effector for repair of DNA double-strand breaks by homologous recombination, a mode of repair which is very important in cell responses to chemotherapy or radiotherapy. In this regard, it is interesting to observe a consistent depletion of the RAD51 protein in NPC xenografts treated with Abexinostat combined to radiotherapy, especially in the C17 model which was the most sensitive to this treatment. One can speculate that assessment of RAD51 depletion in tumor cells after two or three days of combined treatment might be useful for early prediction of tumor responses in NPC patients. For this aim, one would need sensitive and quantitative detection of the RAD51 protein in tumor cell aspirates or brushings, based for example on immuno-cyto-chemistry or imaging mass spectrometry [37].

We also observed that detection of EBER1 by *in situ* hybridization was enhanced in the C15- and C17- PDX under systemic administration of Abexinostat. In contrast, the amount of EBER1 detected by tumor RNA extraction and real time PCR was almost unaffected. EBER1 and 2 are untranslated EBV-encoded RNAs of 167 and 170 nucleotides in length which are consistently and abundantly expressed in malignant cells latently infected by EBV, especially NPC cells [2]. They are suspected to contribute to the malignant phenotype in several manners, including antagonism of interferon pathways and induction of IGF1 production [38]. They are especially abundant in the nucleoplasm where they interact with several proteins like L22 [39], [40]. The discrepancy between *in situ* and RT-PCR detection might be explained by a better accessibility of the hybridizing probe to the target RNA, possibly related to a change in nuclear protein acetylation. In this perspective, it would be interesting to know whether the interactome and functional activity of EBER 1 is modified by Abexinostat. On a translational point of view, in line with the above suggestion concerning quantitative imaging of RAD51 in tumor cells, it might become useful to combine EBER1 with RAD51 detection.

## Supporting Information

Figure S1 **Impact on the tumor growth of C666-1-xeno of three different dose regimens of CDDP used as a single agent.**
(TIF)

Figure S2 **Impact on the tumor growth of C15-PDX of three different dose regimens of CDDP used as a single agent.**
(TIF)

Figure S3 **Impact on the tumor growth of C17-PDX of three different dose regimens of CDDP used as a single agent.**
(TIF)

Figure S4 **Status of the RAD23B protein in xenografted NPC tumors prior to any treatment.**
(TIF)

Figure S5 **Impact of CDDP combined to Abexinostat on the concentration of RAD51 in C17 cells treated** ***ex vivo*** **.**
(TIF)
