# Supplementary figures and images for: Treatment of Nasopharyngeal Carcinoma Cells with the Histone-Deacetylase Inhibitor Abexinostat: Cooperative Effects with Cis-platin and Radiotherapy on Patient-Derived Xenografts
Source: PLoS One. 2014 Mar 11;9(3):e91325. doi: 10.1371/journal.pone.0091325 (PMC3949989; doi:10.1371/journal.pone.0091325)

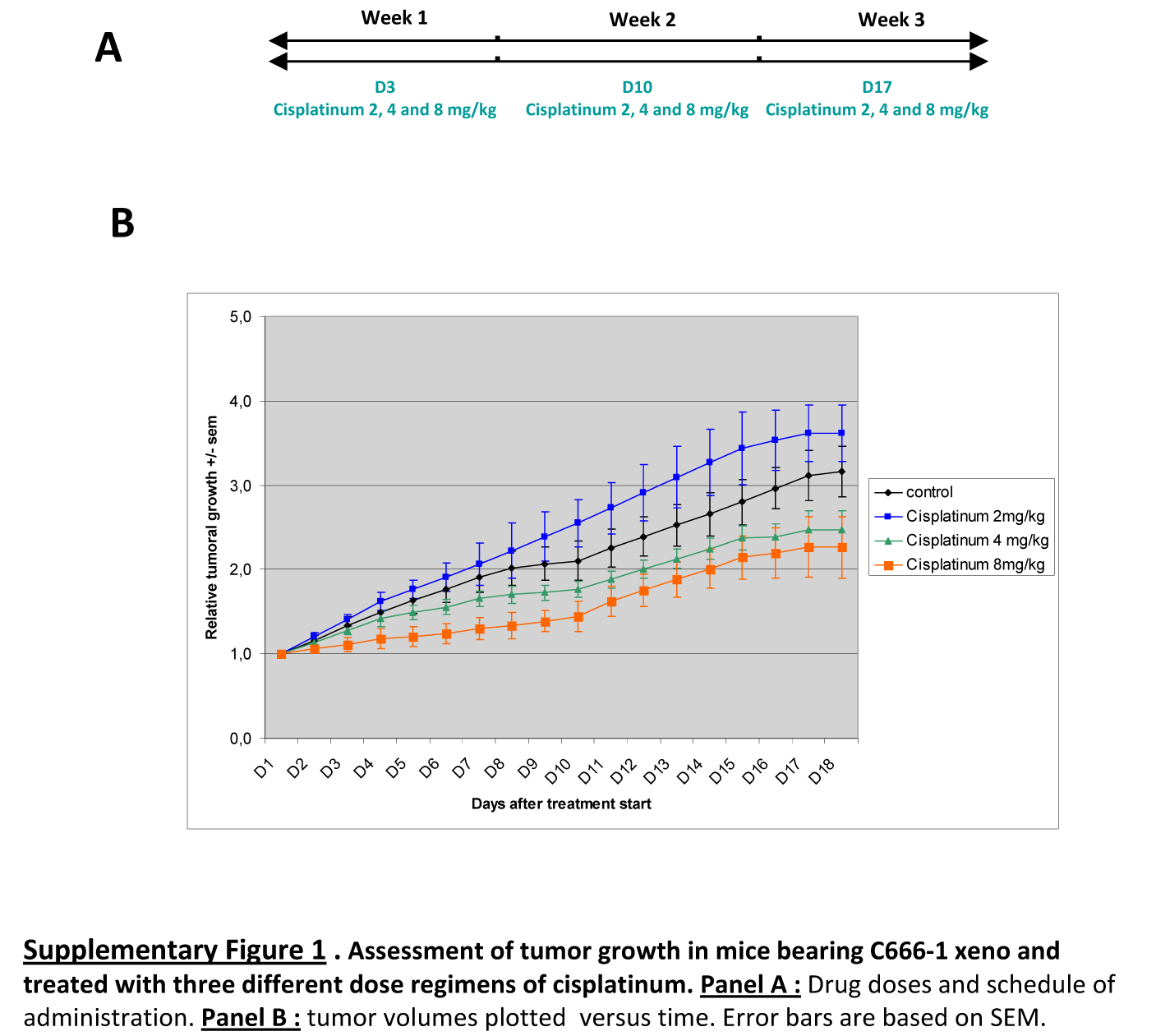

Supplement: Figure S1 — Impact on the tumor growth of C666-1-xeno of three different dose regimens of CDDP used as a single agent. (TIF) [file pone.0091325.s001.tif]

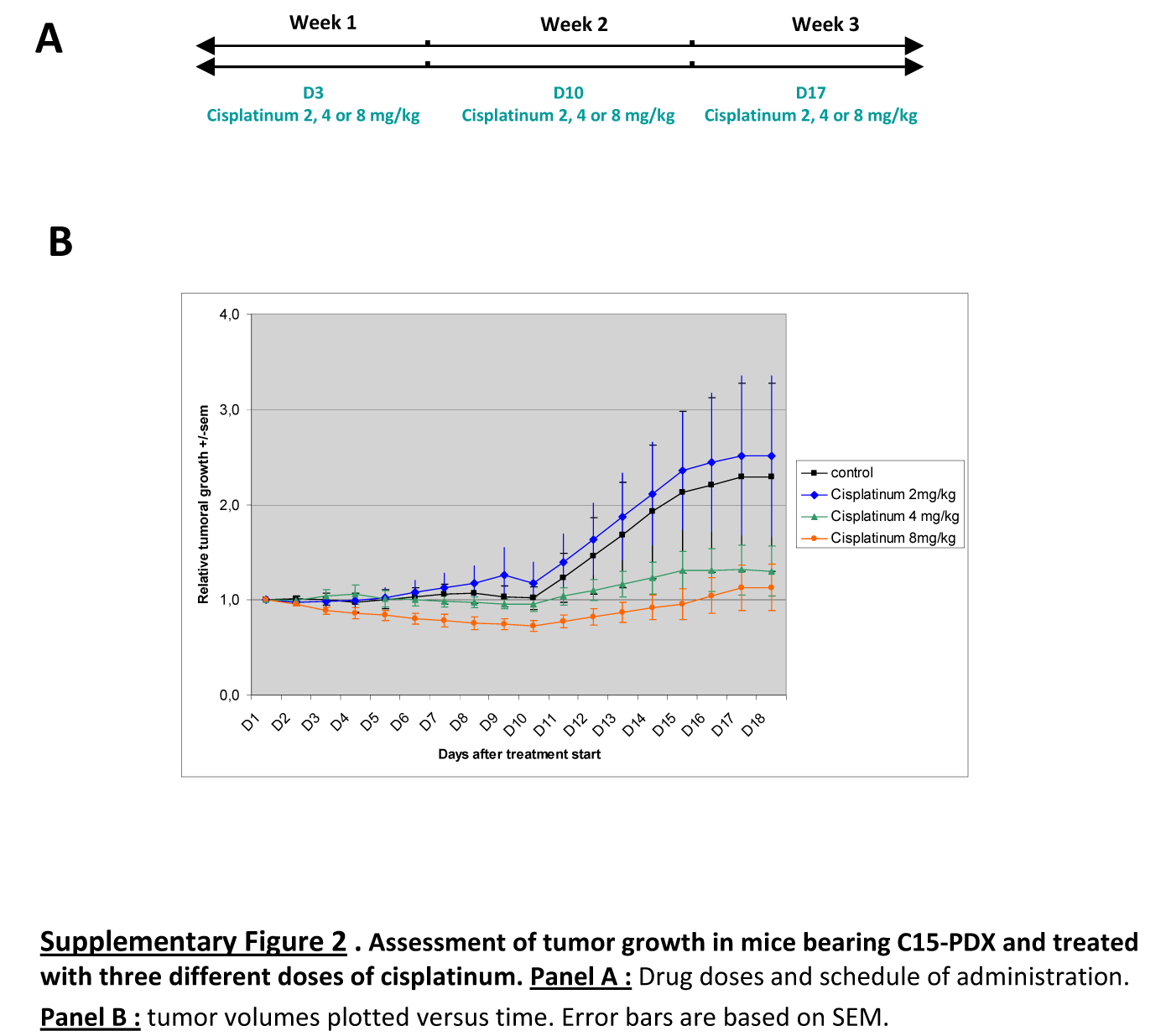

Supplement: Figure S2 — Impact on the tumor growth of C15-PDX of three different dose regimens of CDDP used as a single agent. (TIF) [file pone.0091325.s002.tif]

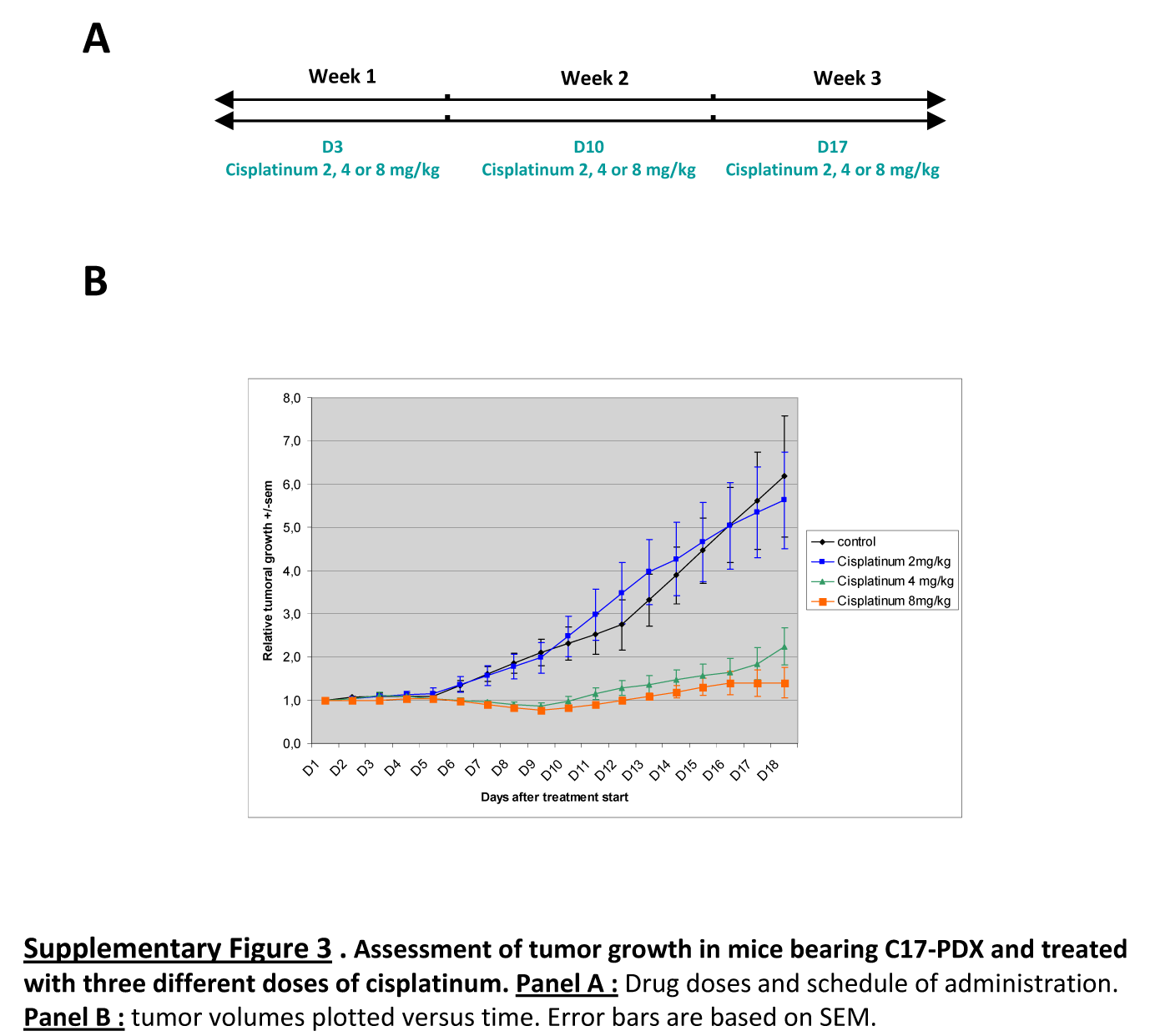

Supplement: Figure S3 — Impact on the tumor growth of C17-PDX of three different dose regimens of CDDP used as a single agent. (TIF) [file pone.0091325.s003.tif]

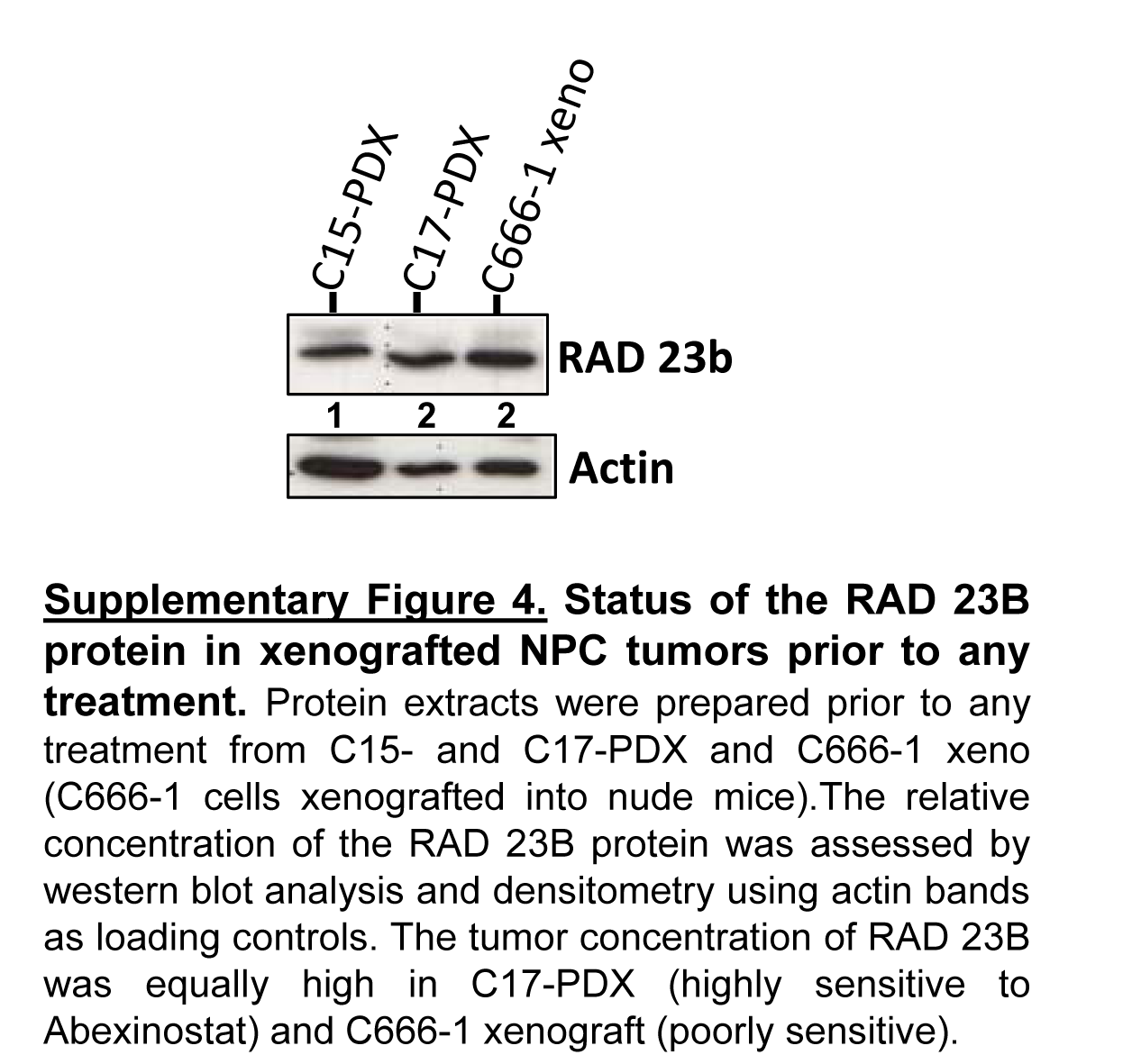

Supplement: Figure S4 — Status of the RAD23B protein in xenografted NPC tumors prior to any treatment. (TIF) [file pone.0091325.s004.tif]

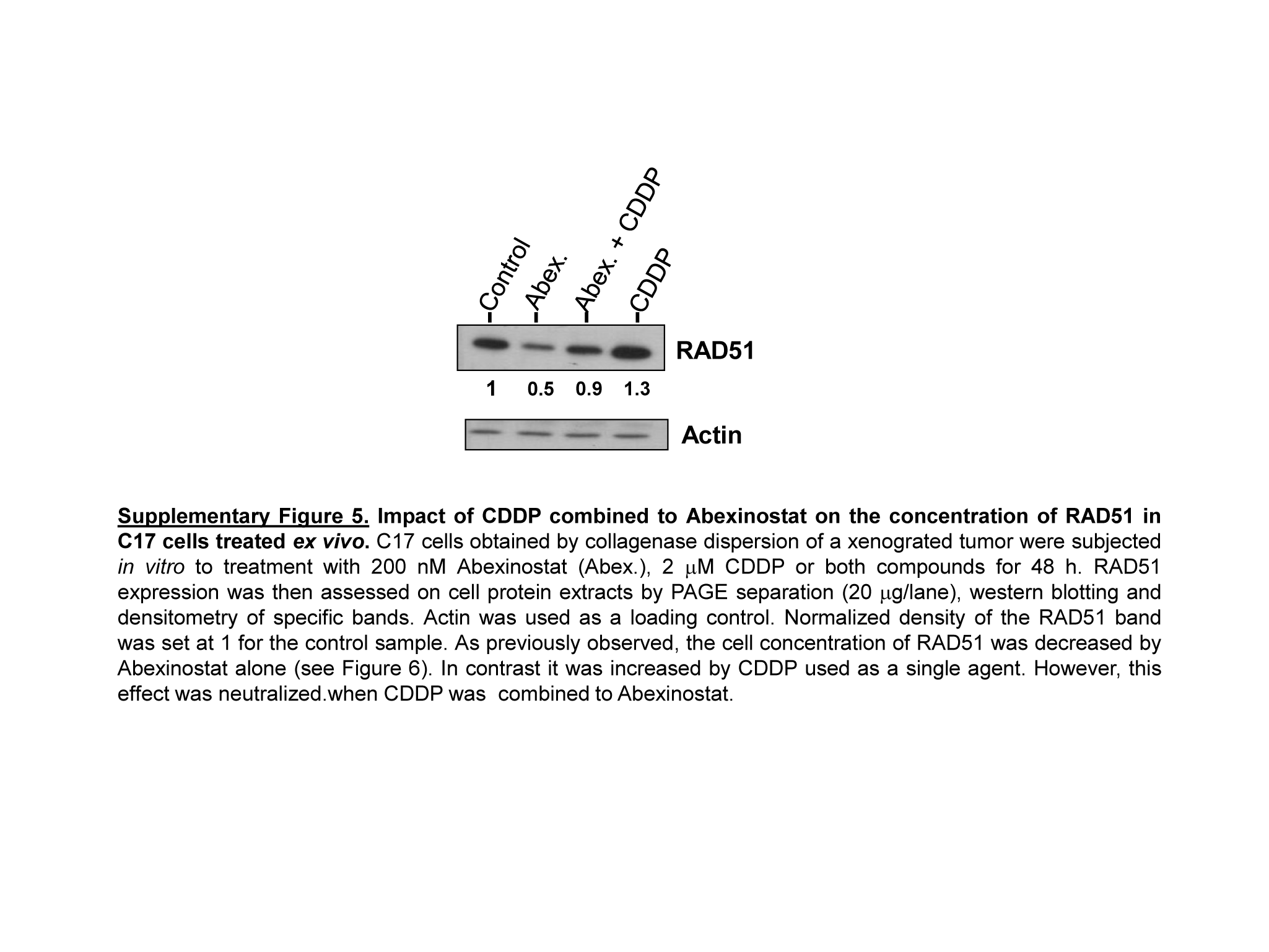

Supplement: Figure S5 — Impact of CDDP combined to Abexinostat on the concentration of RAD51 in C17 cells treated ex vivo . (TIF) [file pone.0091325.s005.tif]
